# Update on Vaccine-Derived Poliovirus Outbreaks — Worldwide, January 2018–June 2019

**DOI:** 10.15585/mmwr.mm6845a4

**Published:** 2019-11-15

**Authors:** Jaume Jorba, Ousmane M. Diop, Jane Iber, Elizabeth Henderson, Kun Zhao, Arshad Quddus, Roland Sutter, John F. Vertefeuille, Jay Wenger, Steven G.F. Wassilak, Mark A. Pallansch, Cara C. Burns

**Affiliations:** ^1^Division of Viral Diseases, National Center for Immunization and Respiratory Diseases, CDC; ^2^Department of Polio Eradication, Detection and Interruption Unit, World Health Organization, Geneva, Switzerland; ^3^Department of Polio Eradication, Research, Policy and Containment Unit, World Health Organization, Geneva, Switzerland; ^4^Global Immunization Division, Center for Global Health, CDC, ^5^Bill and Melinda Gates Foundation, Seattle, Washington.

Certification of global eradication of indigenous wild poliovirus type 2 occurred in 2015 and of type 3 in 2019. Since the launch of the Global Polio Eradication Initiative (GPEI) in 1988 and broad use of live, attenuated oral poliovirus vaccine (OPV), the number of wild poliovirus cases has declined >99.99% (*1*). Genetically divergent vaccine-derived poliovirus* (VDPV) strains can emerge during vaccine use and spread in underimmunized populations, becoming circulating VDPV (cVDPV) strains, and resulting in outbreaks of paralytic poliomyelitis.^†^ In April 2016, all oral polio vaccination switched from trivalent OPV (tOPV; containing vaccine virus types 1, 2, and 3) to bivalent OPV (bOPV; containing types 1 and 3) (*2*). Monovalent type 2 OPV (mOPV2) is used in response campaigns to control type 2 cVDPV (cVDPV2) outbreaks. This report presents data on cVDPV outbreaks detected during January 2018–June 2019 (as of September 30, 2019). Compared with January 2017–June 2018 (*3*), the number of reported cVDPV outbreaks more than tripled, from nine to 29; 25 (86%) of the outbreaks were caused by cVDPV2. The increase in the number of outbreaks in 2019 resulted from VDPV2 both inside and outside of mOPV2 response areas. GPEI is planning future use of a novel type 2 OPV, stabilized to decrease the likelihood of reversion to neurovirulence. However, all countries must maintain high population immunity to decrease the risk for cVDPV emergence. Cessation of all OPV use after certification of polio eradication will eliminate the risk for VDPV emergence.

## Detection of cVDPV1

During January 2018–June 2019, cVDPV type 1 (cVDPV1) circulation was detected in three countries (Indonesia, Myanmar, and Papua New Guinea), compared with one country (Papua New Guinea) during the previous reporting period (*3*). cVDPV1 isolates from acute flaccid paralysis (AFP) cases and environmental surveillance (testing of sewage samples for poliovirus) continued to be detected from the previously reported Papua New Guinea outbreak (*4*) (Table); the AFP patient with the latest case had paralysis onset in October 2018. A new cVDPV1 outbreak was reported in Myanmar; the first patient had paralysis onset in May 2019, and the most recent case occurred in August 2019. A new cVDPV1 outbreak of one case was reported in Indonesia with paralysis onset in November 2018.

**TABLE:** Number of circulating vaccine-derived poliovirus (cVDPV) isolates detected, by serotype, source, and other selected characteristics — worldwide, January 2018–June 2019

Country	Year(s) detected*	Emergence designation^†^	Serotype	No. of isolates from AFP cases	No. of isolates from other human sources (non-AFP)^§^	No. of isolates from environmental (sewage) surveillance	Capsid protein VP1 divergence from Sabin OPV strain^¶^ (%)	2018 estimated national bOPV-3 coverage (%)**	Date of latest outbreak case, healthy child sample, or environmental sample
Angola	2019	HUI-1	2	2	12	0	0.7–1.2	56	Aug 13, 2019
Angola	2019	LNO-1	2	1	1	0	0.8–1.1	56	May 14, 2019
Angola	2019	LNO-2	2	1	0	0	1.1	56	Jul 29, 2019
Benin	2019	JIS-1	2	1	0	0	3.2	75	Jul 11, 2019
Cameroon	2019	JIS-1	2	0	0	1	2.8	78	Apr 20, 2019
CAR	2019	BAM-1	2	2	9	0	1.1–1.3	47	Jun 23, 2019
CAR	2019	BAM-2	2	0	3	0	0.7	47	May 27, 2019
CAR	2019	BIM-1	2	2	1	0	1.0–1.2	47	Jun 29, 2019
CAR	2019	BIM-2	2	0	13	0	1.0–2.0	47	Jun 28, 2019
China	2018–2019	XIN-1	2	1	2	1	1.4–3.7	99	Jun 27, 2019
DRC	2017–2018	HLO-1	2	7	3	0	2.2–3.2	79	Jun 8, 2018
DRC	2018	MON-1	2	11	10	0	2.0–2.9	79	Oct 29, 2018
DRC	2018	HKA-1	2	2	0	0	0.8–0.9	79	Oct 18, 2018
DRC	2019	HLO-2	2	7	1	0	0.9–1.3	79	Sept 9, 2019
DRC	2019	KAS-1	2	1	2	0	0.7–0.8	79	Mar 17, 2019
DRC	2019	KAS-2	2	4	1	0	0.7–1.2	79	Jun 22, 2019
DRC	2019	KAS-3	2	3	0	0	0.9–1.3	79	Jul 13, 2019
DRC	2019	SAN-1	2	6	2	0	0.7–1.4	79	Aug 30, 2019
DRC	2019	TPA-1	2	1	1	0	0.8	79	Aug 14, 2019
Ethiopia	2019	BAN-1	2	1	4	0	5.6	67	Aug 1, 2019
Ghana	2019	JIS-1	2	0	0	1	3.0	98	Sep 3, 2019
Indonesia	2018	PAP-1	1	1	2	0	6.4–6.6	80	Feb 13, 2019
Kenya	2018	BAN-1	2	0	0	2	5.0–5.2	81	Mar 21, 2018
Mozambique	2018	ZAM-2	2	1	2	0	0.7–1.1	80	Dec 17, 2018
Myanmar	2019	KAY-1	1	3	2	0	2.7–3.4	91	Aug 9, 2019
Nigeria	2018–2019	JIS-1	2	45	61	80	1.4–3.7	57	Aug 27, 2019
Nigeria	2019	KGS-1	2	1	0	0	0.9	57	Jul 22, 2019
Nigeria	2019	KGS-2	2	1	0	0	1.1	57	Aug 17, 2019
Nigeria	2018–2019	SOS-3	2	1	0	17	0.7–1.6	57	Mar 24, 2019
Nigeria	2019	SOS-4	2	0	0	3	1.8–2.2	57	Jun 10, 2019
Nigeria	2019	SOS-5	2	1	1	0	1.6–1.7	57	Jun 20, 2019
Niger	2018–2109	JIS-1	2	11	11	0	2.2–2.9	79	Apr 18, 2019
PNG	2018	MOR-1	1	26	8	7	1.4–2.7	67	Nov 4, 2018
Somalia	2017–2019	BAN-1	2	10	1	24	4.2–6.1	47	May 25, 2019
Somalia	2018	BAN-2	3	7	5	12	1.6–2.5	47	Sep 7, 2018
**Total cVDPVs**	**—**	**—**	**—**	161	158	148	**—**	**—**	**—**

**Abbreviations:** AFP = acute flaccid paralysis; bOPV = bivalent oral poliovirus vaccine; CAR = Central African Republic; DRC = Democratic Republic of the Congo; PNG = Papua New Guinea.

* Total years detected for previously reported cVDPV outbreaks (DRC, Kenya, Nigeria, Papua New Guinea, and Somalia).

^†^ Outbreaks list total cases clearly associated with cVDPVs. Emergences indicate independent cVDPV outbreaks and designate the location of the emergence and the number of emergences in a geographic region.

^§^ Contacts and healthy child sampling.

^¶^ Percentage of divergence is estimated from the number of nucleotide differences in the VP1 region from the corresponding parental OPV strain.

** Coverage with 3 doses of OPV, based on 2018 data from the World Health Organization (WHO) Vaccine Preventable Diseases Monitoring System (2018 global summary) and WHO-United Nations Children’s Fund coverage estimates, https://www.who.int/gho/immunization/poliomyelitis/en/. National data might not reflect weaknesses at subnational levels.

## Detection of cVDPV2

**FIGURE 1:**
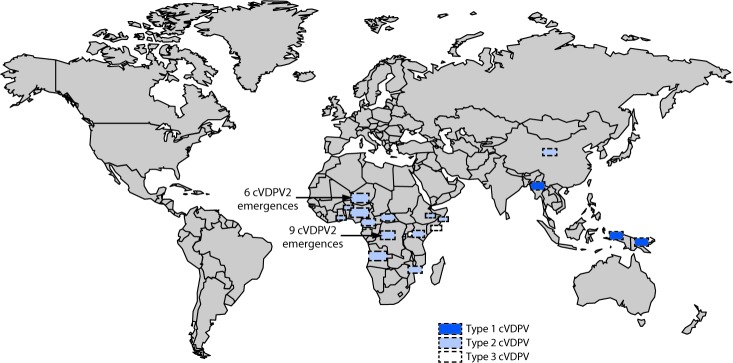
Circulating vaccine-derived poliovirus (cVDPV) outbreaks* — worldwide, January 2018–June 2019 **Abbreviation:** cVDPV2 = circulating type 2 VDPV. * All cVDPV outbreaks were confirmed by genetic sequence data and evolutionary analyses.

**FIGURE 2:**
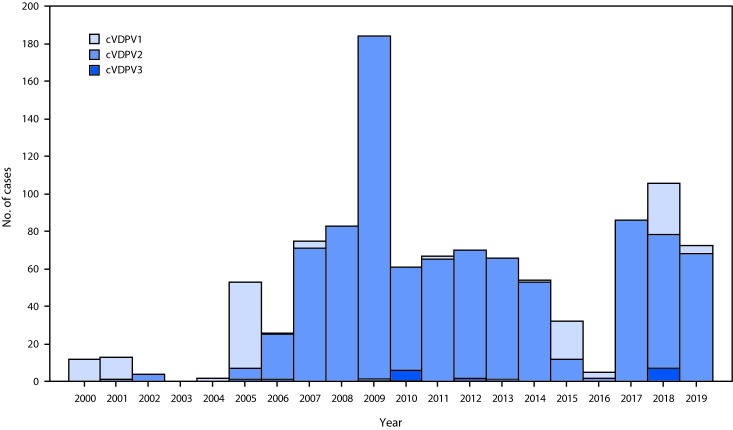
Number of circulating vaccine-derived poliovirus (cVDPV) cases detected, by serotype — worldwide, 2000–2019* **Abbreviations:** cVDPV1 = circulating type 1 VDPV; cVDPV2 = circulating type 2 VDPV; cVDPV3 = circulating type 3 VDPV. * Number of cases detected and reported as of September 10, 2019.

During January 2018–June 2019, 25 cVDPV2 outbreaks were reported in 13 countries (Table). Twelve of the 13 countries were in Africa (Figure 1), and one outbreak occurred in China. During the reporting period, 124 (77%) of the 161 cVDPV cases were cVDPV2, a profile continuing the trend of type 2 dominance that has been observed for the past decade (Figure 2).

**Western Africa**. A single cVDPV2 emergence (designated JIS-1^§^) first detected by environmental surveillance in Jigawa State (Nigeria) in January 2018 was later detected in 12 other states in Nigeria and internationally throughout the reporting period. During the first half of 2019, isolates genetically linked to JIS-1 were detected from AFP cases and environmental surveillance samples initially in Niger, and subsequently in Benin, Cameroon, and Ghana (*5*). Five other independent cVDPV2 emergences were detected in Nigeria: two in Kogi State (KGS-1 and KGS-2) and three in Sokoto (SOS-3, SOS-4, and SOS-5) and Niger (SOS-3) states. During the reporting period, multiple mOPV2 outbreak response activities were conducted in Nigeria (*5*) and in neighboring countries where JIS-1 cVDPV2 was detected.

**Central Africa**. During the reporting period, nine cVDPV2 emergences were detected among 42 AFP cases in five provinces in the Democratic Republic of the Congo (DRC); six of these emergences were detected during the first half of 2019. The previously reported cVDPV2 emergences first detected in Haut Lomami (HLO-1) and Mongala (MON-1) provinces (*6*) and a new 2018 emergence in Haut Katanga (HKA-1) were apparently interrupted (as of September 30, it has been 11–15 months since the latest detection). During January–June 2019, three additional independent cVDPV2 emergences were detected in Kasai (KAS-1–KAS-3) province, and three new cVDPV2 emergences were detected in Haut Lomami (HLO-2), Tshuapa (TPA-1), and Sankuru (SAN-1) provinces.

During the reporting period, four new cVDPV2 emergences were detected in southern districts of Central African Republic (CAR); two were first reported in Bimbo District (BIM-1 and BIM-2) and two were first reported in Bambari District (BAM-1 and BAM-2). As of September 30, six AFP cases were associated with these four cVDPV2 emergences; the first patient had paralysis onset in May 2019. Estimated OPV coverage in CAR both before and after the tOPV-to-bOPV switch has been chronically low (<50%).

Three new cVDPV2 emergences were detected in Angola; two were first detected in Lunda Norte Province (LNO-1 and LNO-2), and one was first detected in Huila (HUI-1) Province. The first cVDPV2 isolate was detected in Lunda Norte from a patient with AFP who had paralysis onset in April 2019. cVDPV2 isolates genetically related to these Angola emergences were later detected from AFP cases in Lunda Sul and Huambo provinces.

**Horn of Africa**. During January 2018–June 2019, cVDPV2 genetically related to the emergence first detected in Somalia in October 2017 (BAN-1) (*6*) was isolated from 10 patients with AFP in Somalia and one in Ethiopia, and from 26 environmental surveillance samples (24 collected in Mogadishu, Somalia, and two in Nairobi, Kenya). BAN-1 cases were detected in provinces in South-Central and Puntland zones of Somalia (Banadeer, Bari, Gedo, Hiran, Lower Juba, Sool, and Togdheer) and in the Somali region of Ethiopia.

**Southern Africa**. During October–December 2018, cVDPV2 was isolated from one AFP patient and two contacts in the Molumbo District of Zambezia Province (Mozambique).

**China**. cVDPV2 was isolated from one environmental surveillance sample collected in Xinjiang province in April 2018 and from one AFP patient and two patient contacts in Sichuan province during May–June 2019. The cVDPV2 sequences from this emergence were 1.4%–3.7% divergent from parental Sabin 2, indicating prolonged circulation.

## Detection of cVDPV3

During 2018, cVDPV3 was isolated from seven patients with AFP (one was coinfected with cVDPV2) and 12 environmental surveillance samples collected in four provinces of Somalia (BAN-2) (Table). The latest patient with AFP had onset of paralysis on September 7, 2018 (*6*).

## Discussion

The number of cVDPV outbreaks detected worldwide increased from nine in six countries during the January 2017–June 2018 reporting period (*3*) to 29 in 15 countries during January 2018–June 2019; 25 (86%) outbreaks were cVDPV2 emergences, 18 (72%) of which were detected during the first half of 2019 in Central and Western Africa. cVDPV2 cases primarily occurred in type 2-naïve children who were born after the switch from tOPV to bOPV and who were therefore at high risk because they were born in areas with chronically low routine and supplementary polio immunization coverage. Seven new cVDPV2 outbreaks were detected in Angola and CAR, countries with no mOPV2 use after the withdrawal of type 2 OPV, but which border DRC, where mOPV2 was used in outbreak responses. Similarly, new cVDPV emergences have occurred in areas of countries that were not part of the mOPV2 response areas (Angola, DRC, and Nigeria). This reflects the increasing susceptibility to type 2 infection and cVDPV2 outbreaks because >3 years have passed since OPV2 cessation. International cVDPV2 spread of JIS-1 from Nigeria to Benin, Cameroon, Ghana, and Niger, and of BAN-1 from Somalia to Ethiopia suggests that multiple mOPV2 responses after detection in each of the countries were of insufficient quality, delayed, or too limited in scope to prevent further spread that, in some cases, led to international transmission.

cVDPV1 and cVDPV3 outbreaks can emerge in countries with suboptimal routine and supplementary immunization coverage; at the subnational level, areas with very wide gaps in immunity carry a higher risk for VDPV emergence and circulation. bOPV campaigns in response to cVDPV1 and cVDPV3 emergences effectively controlled outbreaks in Papua New Guinea (cVDPV1) and Somalia (cVDPV3). cVDPV2 outbreak control requires the use of mOPV2, the release of which depends on the decision of the Director-General of the World Health Organization with the advice from the mOPV2 Advisory Group. Early cVDPV2 detection and timeliness of response are key in addressing circulating VDPV2s; a geographically limited scale mOPV2 campaign should be conducted within 14 days after laboratory cVDPV2 confirmation before larger scale rounds are implemented.

Since April 2016, approximately 300 million mOPV2 doses have been administered in response to cVDPV2 outbreaks (*7*). Although the effective means to stop cVDPV2 outbreaks is mOPV2, the risks associated with its use include seeding of new VDPV2 emergences with the potential for further circulation. The increase in the frequency of new emergences of cVDPV2 outbreaks outside of mOPV2 response areas has led to enhanced surveillance activities and scaling the geographic distribution of mOPV2 campaigns to 1–4 million persons aged <5 years. GPEI partners are providing a surge in technical assistance staffing to outbreak countries to improve the timeliness and quality of mOPV2 responses to aid in more rapid control of outbreaks and limit new emergences. A novel OPV type 2 vaccine, stabilized to decrease the likelihood of reversion to neurovirulence during replication, is in clinical trials (*8*) and, if found to be safe and effective, could be available in limited supply for emergency use as early as mid-2020, and in larger supply at a later date. Expansion of environmental surveillance provides critical indicators for early VDPV detection (*9*); for example, environmental surveillance detection in Cameroon and Ghana in 2019 confirmed circulation of the cVDPV2 emergence of JIS-1 outside Nigeria in the absence of detection of AFP (Cameroon) or before detection (Ghana) of AFP cases.

Since 2000, 1,085 cases of paralysis caused by cVDPV have been reported, 932 (86%) of which were type 2. During the same period, approximately 12 million cases of paralytic polio have been averted through polio eradication efforts. Vaccine-associated paralytic polio can occur in children who receive the vaccine, usually after the first dose, or in their susceptible close contacts, totaling about 2–4 cases per birth cohort of 1,000,000 children before the switch from tOPV to bOPV. Since the switch, an estimated 160–240 cases per year of type 2 vaccine-associated paralytic polio have been averted. In addition, there have been no new cases of VDPV2 excretion identified in persons with primary immunodeficiency (iVDPV) since the switch from tOPV-to-bOPV. Cessation of all OPV use after certification of polio eradication will eliminate the risk for VDPV emergence and spread.

**Summary**

**What is already known about this topic?**

Circulating vaccine-derived polioviruses (cVDPVs) can emerge in settings with low population immunity and cause paralysis.

**What is added by this report?**

Following the synchronized switch from trivalent oral poliovirus vaccine (tOPV, types 1, 2, and 3) to bivalent oral poliovirus vaccine (bOPV, types 1 and 3 only) in 2016, transmission of type 2 cVDPVs was detected in 12 countries in Africa and also in China. Type 1 cVDPVs were identified in Indonesia, Myanmar, and Papua New Guinea, and type 3 cVDPVs were identified in Somalia.

**What are the implications for public health practice?**

All countries must maintain high population immunity. Cessation of all OPV use after certification of polio eradication will eliminate the risk for VDPV emergence.

## Footnotes

* Vaccine-derived poliovirus strains are >1% divergent (for poliovirus types 1 and 3) or >0.6% divergent (for poliovirus type 2) in VP1 sequences from the corresponding OPV strain.

§ Names designate the location of the emergence and the number of emergences in a geographic region; names have been shortened to exclude the country code.
